# Influence of phytochemicals in *piper betle* linn leaf extract on wound healing

**DOI:** 10.1186/s41038-015-0023-7

**Published:** 2015-12-01

**Authors:** Le Thi Lien, Nguyen Thi Tho, Do Minh Ha, Pham Luong Hang, Phan Tuan Nghia, Nguyen Dinh Thang

**Affiliations:** 1 Department of Biochemistry and Plant Physiology, VNU University of Science, Vietnam National University, 334 Nguyen Trai St., Thanh Xuan District, Hanoi, Vietnam; 2 Key laboratory of Enzyme and Protein Technology (KLEPT), VNU University of Science, Vietnam National University, Hanoi, Vietnam

**Keywords:** *Piper betle* Linn, Leaf extract, Wound healing, Malondialdehyde (MDA), Fibroblast (NIH3T3)

## Abstract

**Background:**

Wound healing has being extensively investigated over the world. Healing impairment is caused by many reasons including increasing of free-radicals-mediated damage, delaying in granulation tissue formation, reducing in angiogenesis and decreasing in collagen reorganization. These facts consequently lead to chronic wound healing. *Piper betle* Linn (*Betle*) leaves have been folklore used as an ingredient of drugs for cutaneous wound treatment. However, the effect of *betle leaf* on wound healing is not yet well elucidated. In this study, we aimed to investigate the healing efficacy of methanol leaf extract of *Piper betle* Linn on proliferation of fibroblast NIH3T3 cells as well as full-thickness burn and excision wounds in swiss mice.

**Methods:**

Scratch wound healing assays were conducted to examine the effects of *betle* leaf extract on healing activity of fibroblast cells. Burn and excision wounds on swiss mouse skins were created for investigating the wound healing progress caused by the *betle* leaf extract. Malondialdehyde (MDA) was also evaluated to examine the products of lipid hydroperoxide (LPO) under conditions of with or without *betle* leaf extract treatment.

**Results:**

The results of this study showed that *Piper betle* Linn leaf extract in methanol increased proliferation of NIH3T3 cells and promoted wound healing *in vitro* and *in vivo* with both burn wound and excision wound models. In addition, this extract significant decreased level of malondialdehyde (MDA) in liver of treated-mice compared with that in non-treated mice.

**Conclusions:**

Our results suggest that *Piper betle* Linn can be used as an ingredient in developing natural origin drugs for treatment of cutaneous wounds.

## Background

The skin is one of the largest organs and plays important biological roles in the body. Skin contributes in maintaining the fluid homeostasis, regulating the thermo effects, sending sensory signals to the brain, and metabolizing various substances [1, 2]. The skin is also the first barrier to protect the body against the infections from the environment. Any damage of this physical barrier may lead to attacks of pathogens and consequently cause infection in the body [1, 2]. Healing impairment is caused by many reasons including increasing in free-radicals-mediated damage, delaying in granulation tissue formation, reducing in angiogenesis and decreasing in collagen reorganization. These facts consequently led to chronic wound healing [3, 4].

Several plants and their products are used in folk medicine to treat wounds [4–8]. *Piper betle* Linn is a member of *Piperaceae* family and to be cultivated in most of South and Southeast Asia including Vietnam. It could be used as an ingredient in drugs because of its medicinal properties. *Betle* leaves contain main chemical components such as betal-phenol, chavicol and other phenolic compounds. These components might give strong potentials in anti-fungi, anti-bacteria properties of *betle* [9]. *Betle* leaf also has been reported that it exhibit potentials in wound healing treatment and other diseases [10–16]. However, the effect of *betle* leaf on wound healing is not yet well examined.

Topical anti-bacterial agents and disinfectants are good in protecting against infection; however these agents may cause the allergic reactions and skin irritations and result in the rate of skin regeneration and increases the recovery time [17, 18]. Although there are modern methods such as recombinant growth factors and tissue-engineered wound dressings used for wound treatments, they are so expensive for patients in the low-income countries. Therefore, in developing countries, many drugs originated from medicinal plants are being used as alternative and complementary systems of medicines to treat wounds and several wound healing processing diseases [4–8].

Hence, the present study was undertaken to examine the influence of methanol *betle* leaf extract on proliferation of fibroblast NIH3T3 cells and healing of burn and excision wounds in swiss mice.

## Methods

### Plant material

*Piper betle* Linn (*Betle*) leaves were collected from Vietnam Pharmacy Institute in September 2013 and identified at the Department of Biochemistry and Plant physiology, Faculty of Biology, VNU University of Science, Vietnam National University, Hanoi, Vietnam.

### Preparation of leaf extract

**Fig. 1:**
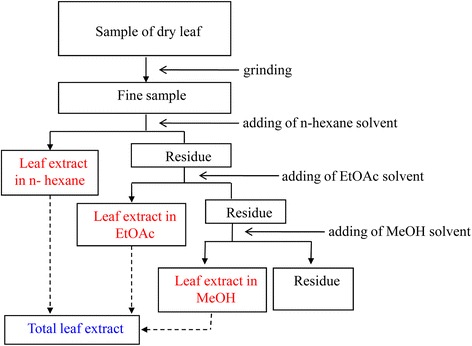
The scheme of leaf extraction procedure with three types of organic solvents including n-hexane, EtOAc and Methanol

Fresh leaves of *betle* were cleaned and washed thoroughly with water and re-washed with distilled water. Washed fresh leaves were shade dried, powdered mechanically, and sieved by using a mesh. In the preparation of organic solvent extracts, 5 g of powdered material was refluxed with 1/10 w/v in a soxhlet apparatus for an hour. The extract was filtered, and the solvent was removed under reduced pressure at 40 ± 5 °C using a rotary flash evaporator. The scheme of the extraction procedure was presented in the Fig. 1 and the effectiveness of extraction procedure was presented in the Table 1. We used simultaneously three types of organic solvents including n-hexane, EtOAc and MeOH with gradually increasing in polarities to extract substances in the leaves of *betle*. Normally, substances which could be dissolved in methanol (alcohol) have high biological and pharmaceutical activities. Therefore, in this study we focused on investigate the effect of methanol extract on wound healing.

**Table 1:** Consecutive extraction efficiency of phytochemicals from *Polygonum multiflorum* in three different solvents including n-Hexane, EtOAc and MeOH

Name	Total wet weight (gram)	Total dry weight (gram)	Dry extract weight (gram)/10g dry weight		
			n-Hexane	EtOAc	MeOH
*Piper betle* L.	400	77.6	1.07	2.89	1.40

### Drug formulations

Drug formulation was prepared from methanol extract. For topical administration, 5 g of the methanol extracts was separately incorporated with 100 g of 2 % sodium alginate to get 5 % w/w gel. The drug formulation was prepared every fourth day. The drug was daily administered on mice.

### Cell culture

Fibroblasts (NIH3T3) (Riken Bioresource Center, Japan) was cultured in DMEM with 10 % FBS supplemented with 10 % Fetal Bovine Serum (FBS) and 1 % Penicillin/Streptomycin at 37 °C in 5 % CO_2_ on collagen-coated dishes [19].

### Crystal violet assay

Crystal violet assay was performed using the method previously described in our reports [20, 21]. Briefly, cells (3 × 10^4^ cells) were plated in six-well plates and cultured for 24 h. Cells were then treated with or without methanol extract of *betle* and cultured for further 3 days. The viable adherent cells were fixed with 10 % formalin and stained with 0.1 % crystal violet. Absorbance at 595 nm in the stained cells solubilized with 0.1 % SDS was measured using a microplate reader.

### Scratch wounding assay

Cell wound healing was performed as described previously studies [20, 22]. Six-well plates were incubated overnight in 1 mL of RPMI 1640 media containing collagen at 40 μg/ml. Cells were cultured in RPMI 1640 (10 % FBS) in the collagen-coated plate until the cell confluence reached to more than 90 %. Scratch wounds were created in confluent monolayers using a sterile p200 pipette tip. After that, cell plate was washed three times with PBS to remove the suspended cells. Then, the wounded monolayers were cultured in RPMI 1640 medium. After incubation for 24 h, repopulations of the wounded areas were observed under microscope (OLYMPUS). The migration distances of cells into the scratching areas were measured and calculated.

### Animals

Swiss mice weighing 15–20 g were purchased from Vietnam National Institute of Hygiene and Epidemiology, Hanoi and maintained at standard housing conditions. The animals were fed with a commercial diet [23] (also from the Vietnam National Institute of Hygiene and Epidemiology) and water. The Vietnam National University Ethical Committee permitted the study.

### Evaluation of wound healing activity

For the assessment of the wound-healing activity, burn and excision wound models were used [24, 25]. Three groups, each containing five animals, including control group (group I), burn wound group (group II) and excision wound group (group III) were used. Fifty milligrams (50 mg) of the formulated drug was topically applied for each animal once a day. The Group I animals were treated by topical applications of 5 % w/w gel, while the Groups II and III animals were treated with ethanol extracts. The animals were anesthetized as described by Morton and Malone [24] using diethylether (C_2_H_5_OC_2_H_5_). For the excision wound model, the skin of the impressed area of mouse was excised to full thickness to obtain a wound area of about 120 mm^2^ [24]. For burn wound model, full-thickness burn wound was created by using an aluminum metal rod (diameter 120 mm × 100 mm) heated to 90 °C. Hot rod was exposed on the shaved area in the skin of mouse for 20 s. The drug was topically applied once a day till complete epithelialization, starting from the day of the operation. Then, wound areas and wound epithelizations were measured at regular intervals of time to see the percentage of wound closure and formation of new epithelial tissues. The percentages of wound closure were recorded on days 2, 4, 6 and 3, 7, 15 for burn wounds and excision wounds, respectively.

### MDA analysis

After wounds were totally healed, mice were anesthetized by intraperitoneal injection of ketamine and sacrificed. Liver and soft leg tissues were collected and stored as frozen tissues in liquid nitrogen for biochemically MDA analysis. Tissue samples (300 mg for each) were homogenized in ice-cold tamponade containing 150 mM KCl for determination of MDA. MDA levels were assayed for products of lipid hydroperoxide (LPO). MDA was measured with thiobarbituric acid at 532 nm using a spectrofluorometer, as described previously [26, 27].

### Statistical analysis

In this study, all experiments were repeated three times and collected data were statistical analysis with suitable methods. To compare two groups, Mann–Whitney *U*-test was used in case of non-parametric and Student’s *t*-test was used for parametric. To compare multi groups with control group, one-way ANOVA analysis and Dunnett post-hoc test were used. To assess the combination effect of two factors, two-way ANOVA with replication analysis and Tukey’s HSD post-hoc test were used. The significant differences were set at three levels with *P* < 0.05 [20].

## Results

### Betle promoted proliferation of fibroblast NIH3T3 cells

**Fig. 2:**
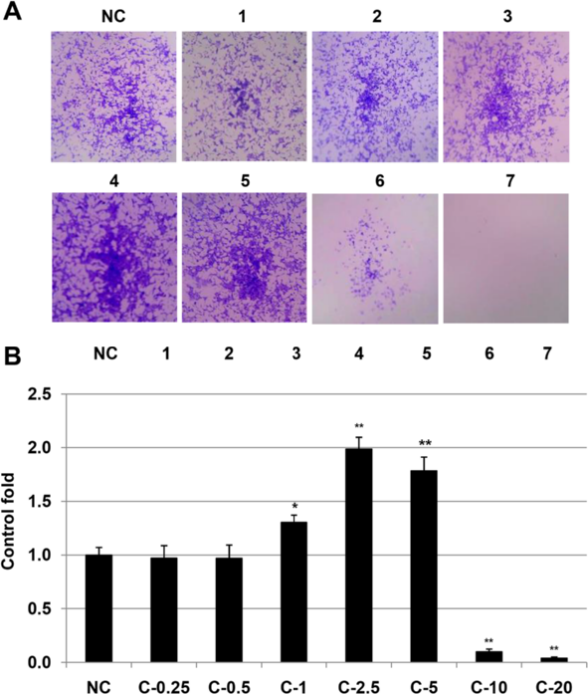
Effect of methanol leaf extract at various concentrations on proliferation of NIH3T3 cells were presented in photos (**a**) and a graph (**b**). Leaf extract concentration tested including: 0.25 μg/mL (lane 1), 0.5 μg/mL (lane 2), 1.0 μg/mL (lane 3), 2.5 μg/mL (lane 4), 5.0 μg/mL (lane 5), 10.0 μg/mL (lane 6), 20.0 μg/mL (lane7). * and **Significantly different (*p* < 0.05, and 0.01, respectively) from the control by the one-way ANOVA analysis and Dunett post-hoc test

NIH3T3 cells were treated with *betle* at various concentrations (0, 0.25, 0.5, 1.0, 2.5, 5.0, 10 and 20 μg/mL). The results were presented in photos (Fig. 2a) and a graph (Fig. 2b). At the low concentrations of 0.5 and 1.0 μg/mL, *betle* had no effect on proliferation of NHI3T3. However, at high concentrations of 10 and 20 μg/mL, *betle* was so high toxic to NHI3T3 cells with more than 90 % and 99 % cells dead, respectively. Interestingly, at concentrations 1.0, 2.5 and 5.0 μg/mL, *betle* significantly increased proliferation of NIH3T3 cells up to 1.31, 2.0 and 1.78 folds compared with non-treated cells, respectively. Fibroblast NIH3T3 cells play important role in collagen synthesis and collagen formation in skin tissue during wound healing process [28], therefore our results suggested that *betle* at suitable concentrations was able to increase of NIH3T3 cell proliferation and consequently it may promote the rate of wound healing.

### Betle increased healing rate of scratch wound in vitro

**Fig. 3:**
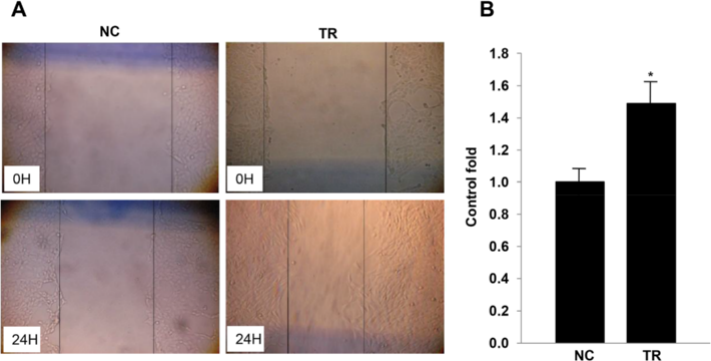
Effect of methanol leaf extract on scratch wound healing of NIH3T3 cells were presented in photos (**a**) and a graph (**b**). Leaf extract concentration tested including: 0.0 μg/mL, and 5.0 μg/mL. * and **Significantly different (*p* < 0.05, and 0.01, respectively) from the control by the Mann–Whitney *U*-test

We next investigated the healing rate of NIH3T3 by scratch wound healing assay. NHI3T3 were cultured in starving medium (0.5 % FBS) for 8 h before treated or/and untreated with *betle* at concentration of 5 μg/mL for 2 days untill the cell population reached up to more than 90 % confluence. Then, scratch wound healings were examined within 24 h. Healing activities of NIH3T3 cells were presented in the Fig. 3. Our result showed that healing velocity of NIH3T3 with *betle* treatment was 1.53 fold higher than that of untreated cells. The migration of cells into the wounds plays key role in wound healing process.

### Betle increased healing rate of burn wounds in swiss mice

**Fig. 4:**
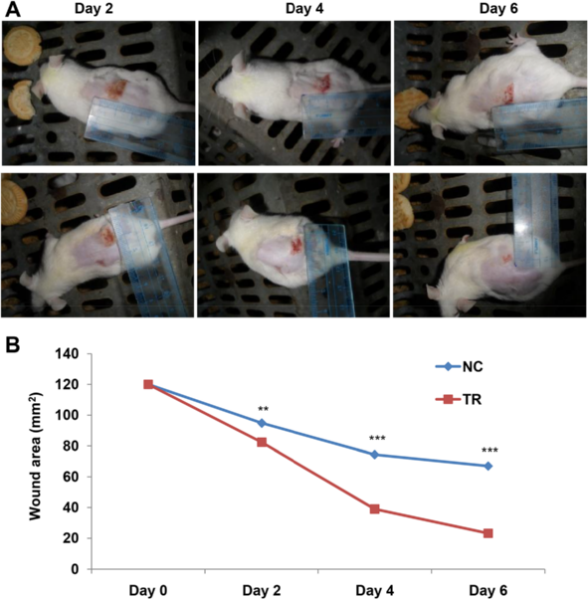
Effects of methanol leaf extract (5 % w/w gel) on burn wound healing were presented in photos (**a**) and a graph (**b**). ** and ***Significantly different (*p* < 0.01, and 0.001, respectively) from the control by two-way ANOVA with replication analysis and Tukey’s HSD post-hoc test

Burn wounds (120 mm^2^) were created by the hot metal rod on the back skins of the mice and topical treated with drug daily for 7 days. The wound areas were observed and measured for every two days. The results were presented in photos (Fig. 4a) and a graph (Fig. 4b). It showed that 5 % *betle* drug gel had good effect in healing rate of the burn wounds. The average percentages of burn healing areas were 32, 68 and 80 in *betle*-treated mice and 20, 38 and 43 in control mice at the 2^nd^, 4^th^ and the 6^th^ days post-wounding, respectively.

### Betle increased healing rate of excision wounds in swiss mice

**Fig. 5:**
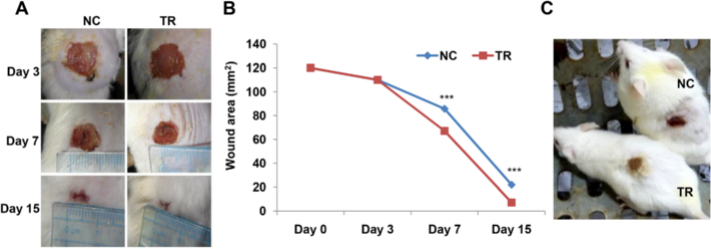
Effects of methanol leaf extract (5 % w/w gel) on excision wound healing were presented in photos (**a**, **c**) and a graph (**b**). ***, Significantly different (*p* < 0.001) from the control by two-way ANOVA with replication analysis and Tukey’s HSD post-hoc test

The impressed areas of the back skins of swiss mice were excised to full thickness to obtain wound areas of 120 mm^2^. Wounds were daily topical administrated with drug for 15 days and the areas of wounds were measured at the third, the seventh and the fifteenth days post-wounding. The results were presented in photos and in a graph in the Fig. 5. Our results indicated that the 5 % *betle* drug gel had positive effect on excision wound healing in swiss mice. At the third day post-wounding, there were no difference in the rates of wound healing between *betle*-treated mice and control mice, however at the seventh day and fifteenth day there were significant differences in the rates of wound healing between *betle*-treated mice (TR) and control mice (NC). The average percentages of wound healing areas at the seventh day and fifteenth day were 44 and 94 for *betle*-treated mice and 28 and 82 for control mice, respectively. Wound healing is related to two processes including wound contraction and wound re-epitheliazation [3]. However, because of loose skin of rodents, wound closure might be caused mainly by wound contraction. Therefore it was quite hard to clarify the effect of drug on the wound re-epitheliazation. Thus, we decided to create wounds with large areas (225 mm^2^) for sure that it will take a long time for skin contraction in order that we can observe wound healing by re-epithelialization. Our results showed that at the 12th day post wounding, re-epitheliazation of the wounds on the skin of the *Piper betle-*treated mice were better than that of the control mice. While the control wounds were still in concave forms with red colors, the *Piper betle-*treated wounds were filled with formation of dry scales (Fig. 5c). This result suggested that drug gel was not only able to increase skin contraction but also to promote re-epitheliazation of the wounds.

### Betle decreased activity of MDA

**Fig. 6:**
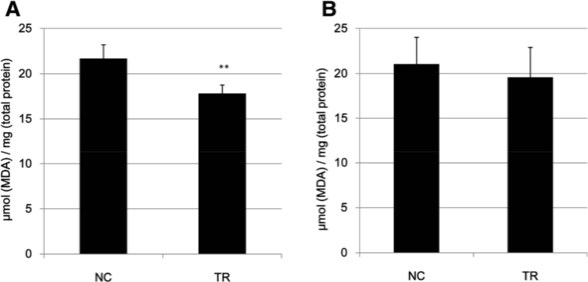
Effects of leaf extract (5 % w/w gel) on concentrations of malondialdehyde (MDA) in liver tissues (**a**) and leg soft tissues (**b**) of non-treated (NC) and *betle*-treated mices were examined by MDA analysis. **, Significantly different (*p* < 0.01) from the control by Student’s *t*-test

In generally, natural leaf extracts contain many polyphenolic compounds acting as antioxidant agents [29]. Lipid hydroperoxides (LPO) are products from oxidative damage in lipid-containing structures in cells [30]. Malondialdehyde (MDA) is considered as an important marker for LPO [26]. The high level of MDA produced in the tissues indicates that the tissues might be injured and DNA might be damaged. In the other hand, the excessive MDA could bind to free amino groups of proteins and result in the formation of MDA-modified protein adducts [26]. Therefore we decided to examine the effect of *betle* leaf extract on the levels of MDA in liver and leg soft tissues of mice. Our results showed that levels of MDA in liver and leg soft tissues of *betle*-treated mice were 18 % and 7 % lower than that of non-treated mice, respectively (Fig. 6).

## Discussion

In this study, we consecutively used three organic solvents with gradually increase in polarity including n-hexane, ethylacetate and methanol to extract phytochemicals in the *betle*. In generally, the two first solvents of n-hexane and ethylacetate are used to eliminate of substances which have non- or low- biological activities from the samples before extracting in methanol to collect substances which have biological activities. And then, we had tried to investigate the effects of not only methanol extract but also n-hexane and ethylacetate extracts on NIH3T3 cells, however, it was not showed the impressive effects of n-hexane and ethylacetate extracts on the cells (data not shown).

However, our *in vitro* results of this study showed that at the concentrations of 2.5-5 μg/mL *betle* leaf extract in methanol had positive effects on proliferation of NIH3T3 cells. Basing on this, we chose the concentration of 5 % (v/v) of drug gel for *in vivo* experiments but not perform a real dose-dependent curve to obtain EC50 and EC05. Because our methanol extract used in this study was still in crude form (i.e., there may have many substances in the extract). It was not so important to determine the very precise concentration of the crude extract; however this kind of experiment should be done in case of using single substance. Therefore, we followed the previous reports [1, 2, 5, 6] to select the concentration of extract for *in vivo* experiments as about 10 times higher than that used for *in vitro* experiments (5 % V/V).

Our *in vivo* experiments revealed that *betle* leaf extract gel significantly increased healing rates of burn wounds and excision wounds in swiss mice. Moreover, statistical results by two-way Anova with replication analysis and Tukey’s HSD post-hoc test showed that burn wound healing were affected by both leaf extract and treatment time and there were interaction between these two factors in both cases of burn wound (Fig. 4) and excision wound (Fig. 5). In the case of burn wound, it was also indicated that *betle* leaf extract had the strongest effect on wound healing in the period of 2–4 days post wound making (PWM), followed by periods of 0–2 days PWM the smallest effect in the period of 4–6 days PWM with significant differences (Table 2). In the case of excision wound, in the period of 0–3 days PWM, there was no difference in wound healing rate between control groups and treated groups; however, at the period of 3–7 days PWM, the effect of *betle* leaf extract was strongest and followed by period of 7–15 days PWM with significant differences (Table 3). These statistic results may suggest a hint that we can screen various leaf extracts on wound healing activities and determine the wound healing effects of these extracts at different periods during treatment times to develop an optimum clinical protocol by changing or combining of these various leaf extracts.

**Table 2:** Two-way Anova with replication analysis and Tukey’s HSD post-hoc test showed combination effects of leaf extract and treatment time on burn wound healing

	Day 0	Day 2	Line segment slopes	Absolute difference between slopes
NC	120	94.8	−25.2	12.4
TR	120	82.4	−37.6	
	Day 2	Day 4	Line segment slopes	Absolute difference between slopes
NC	94.8	74.4	−20.4	23.0
TR	82.4	39	−43.4	
	Day 4	Day 6	Line segment slopes	Absolute difference between slopes
NC	74.4	67	−7.4	8.4
TR	39	23.2	−15.8

**Table 3:** Two-way Anova with replication analysis and Tukey’s HSD post-hoc test showed combination effects of extract and treatment time on excision wound healing

	Day 0	Day 3	Line segment slope	Absolute difference between slopes
NC	120	110	−10	0
TR	120	110	−10	
	Day 3	Day 7	Line segment slope	Absolute difference between slopes
NC	110	85.8	−34.2	18.6
TR	110	67.2	−52.8	
	Day 7	Day 15	Line segment slopes	Absolute difference between slopes
NC	85.8	22	−63.8	3.8
TR	67.2	7.2	−60	

## Conclusions

In this preliminary research, we also found that *betle* can induce proliferation of fibroblast cells and promote wound healing in swiss mice but not caused to any strange behavior or phenomenon of treated mice. Moreover, it was revealed the fact that *betle* promoted wound healing progression with lowering levels of MDA in liver and leg soft tissues of treated mice.

Taken together, our results suggest that *Piper betle* can be used in alternative and complementary systems to improve efficacy of medicines used for cutaneous wound treatment.
